# Downregulation of PERK activity and eIF2α serine 51 phosphorylation by mTOR complex 1 elicits pro-oxidant and pro-death effects in tuberous sclerosis-deficient cells

**DOI:** 10.1038/s41419-018-0326-2

**Published:** 2018-02-15

**Authors:** Jothilatha Krishnamoorthy, Clara Tenkerian, Jyotsana Gupta, Nour Ghaddar, Shuo Wang, Cedric Darini, Kirk A. Staschke, Abhishek Ghosh, Valentina Gandin, Ivan Topisirovic, Arnold S. Kristof, Maria Hatzoglou, George Simos, Antonis E. Koromilas

**Affiliations:** 1 Lady Davis Institute for Medical Research, McGill University, Sir Mortimer B. Davis-Jewish General Hospital, Montreal, QC H3T 1E2 Canada; 2 Oncology Research Division, Lilly Research Laboratories, Indianapolis, IN 46285-0428 USA; 3 Department of Oncology, Faculty of Medicine, McGill University, Montreal, QC H2W 1S6 Canada; 4 Department of Critical Care, McGill University Health Centre and Meakins-Christie Laboratories, Department of Medicine, McGill University, Montreal, QC H3A 1A1 Canada; 5 Department of Genetics, Case Western Reserve University, Cleveland, OH 44106 USA; 6 Laboratory of Biochemistry, School of Medicine, University of Thessaly, Larissa, 41110 Greece

## Abstract

Oxidative stress determines cell fate through several mechanisms, among which regulation of mRNA translation by the phosphorylation of the alpha (α) subunit of the translation initiation factor eIF2α at serine 51 (eIF2αP) plays a prominent role. Increased eIF2αP can contribute to tumor progression as well as tumor suppression. While eIF2αP is increased in most cells to promote survival and adaptation to different forms of stress, we demonstrate that eIF2αP is reduced in tuberous sclerosis complex 2 (TSC2)-deficient cells subjected to oxidative insults. Decreased eIF2αP in TSC2-deficient cells depends on reactive oxygen species (ROS) production and is associated with a reduced activity of the endoplasmic reticulum (ER)-resident kinase PERK owing to the hyper-activation of the mammalian target of rapamycin complex 1 (mTORC1). Downregulation of PERK activity and eIF2αP is accompanied by increased ROS production and enhanced susceptibility of TSC2-deficient cells to extrinsic pro-oxidant stress. The decreased levels of eIF2αP delay tumor formation of TSC2-deficient cells in immune deficient mice, an effect that is significantly alleviated in mice subjected to an anti-oxidant diet. Our findings reveal a previously unidentified connection between mTORC1 and eIF2αP in TSC2-deficient cells with potential implications in tumor suppression in response to oxidative insults.

## Introduction

Oxidative stress occurs when the equilibrium between cellular production of pro-oxidants and anti-oxidant defense mechanisms is disrupted leading to accumulation of reactive oxygen species (ROS), including the superoxide radical O_2_^∙−^, hydrogen peroxide H_2_O_2_, and the highly reactive hydroxyl radical ∙OH^1^. Cells respond to ROS by increasing the expression of anti-oxidant genes as well as by activating pathways that control survival and adaptation to oxidative stress^2^. An important pathway induced by oxidative stress involves the activation of phosphatidylinositol 3-kinase (PI3K) owing to inactivation of the phosphatase and tensin homolog deleted in chromosome 10 (PTEN) and/or activation of the adaptor P66^Shc^^2–4^. Increased PI3K activity leads to the activation of AKT/protein kinase B (PKB), which in principle promotes survival but can also lead to premature senescence or death under conditions of severe oxidative stress^4, 5^. Downstream of AKT, the mammalian target of rapamycin complex 1 (mTORC1) integrates intracellular and extracellular cues, including growth factors, amino acids, oxygen, energy status and stress, to regulate several major cellular processes such as protein synthesis and autophagy^6^. The activity of mTORC1 can be both positively and negatively regulated by oxidative stress depending on ROS levels and time of exposure to this form of stress^7^. mTORC1 is negatively regulated by the tuberous sclerosis complex (TSC), which consists of TSC1 (hamartin), TSC2 (tuberin) and Tre2-Bub2-Cdc16-1 domain family member 7 (TBC1D7) and acts as an inhibitory GTPase-activating protein for the small GTPase RAS homolog enriched in brain (RHEB)^8–10^. Cells impaired for TSC expression or localization to peroxisomes exhibit increased mTORC1 activity under oxidative stress, which is associated with decreased autophagy and increased cell death^11, 12^.

An immediate response of cells to oxidative stress is the general inhibition of mRNA translation to retain the equilibrium between protein synthesis and clearance, and to maintain homeostasis^13^. Among the different translation inhibitory mechanisms, phosphorylation of the α subunit of the eukaryotic translation initiation factor eIF2 at serine 51 (herein referred to as eIF2αP) plays a prominent role^14^. eIF2α is phosphorylated by a family of four serine–threonine kinases, namely heme-regulated inhibitor (HRI), protein kinase double-stranded (ds) RNA-dependent (PKR), PKR-like endoplasmic reticulum (ER) resident kinase (PERK), and general control non-repressible-2 (GCN2)^14, 15^. Each kinase is activated by distinct forms of stress, a process termed the integrated stress response (ISR)^14, 15^. Increased eIF2αP results in a severe attenuation of de novo protein synthesis but at the same time promotes translation of select mRNAs like those encoding for the activating transcription factors 4 (ATF4) and ATF5 in mammalian cells, which contribute to adaptive homeostasis^16, 17^.

Increased eIF2αP plays an important role in the regulation of redox homeostasis and adaptation to oxidative stress^18–20^. Oxidative stress is linked to ER stress given that accumulation of misfolded proteins in the ER leads to generation of ROS, whose deleterious effects are counterbalanced by the induction of the unfolded protein response (UPR)^1^. UPR activates the PERK-eIF2αP arm, which via the translational upregulation of ATF4 results in the transcriptional induction of genes encoding anti-oxidant proteins^18, 19, 21^. The anti-oxidant function of eIF2αP further involves the attenuation of general protein synthesis, which decreases client protein load and prevents illegitimate disulfide bond formation in the ER leading to a sufficient amount of reducing equivalents to alleviate cells from oxidative stress^22^. Also, attenuation of protein synthesis by increased eIF2αP prevents ATP depletion, stimulation of mitochondrial oxidative phosphorylation and ROS production^23^.

Inactivation of either PERK or eIF2αP in mouse or human primary fibroblasts is associated with increased ROS synthesis and premature senescence^18–20^. On the other hand, immortalized and tumor cells impaired for eIF2αP are tolerant to intrinsic ROS but become highly sensitive to extrinsic oxidative insults^20^. Increased eIF2αP promotes cell adaptation to oxidative stress via the translational upregulation of ATF4 and expression of anti-oxidant genes^18, 19^. Also, increased eIF2αP determines AKT’s function under oxidative stress, which can increase tolerance to oxidative stress or promote death under excessive oxidative stress^4, 24, 25^.

We investigated the role of eIF2αP in TSC2-deficient cells, which are impaired for PI3K-AKT signaling and hyper-active for mTORC1. We found that TSC2-deficient mouse and human cells contain increased ROS and are highly sensitive to oxidative insults. This process is associated with the downregulation of the PERK-eIF2αP arm owing to mTORC1 hyper-activation. We also found that decreased eIF2αP elicits pro-oxidant and pro-death effects in TSC2-deficient cells and is associated with an inhibition of tumor initiation in immune deficient mice. Our work shows that eIF2αP is a nodal point of mTORC1 signaling, which can determine the susceptibility of TSC2-deficient cells to oxidative insults and their efficacy in tumor formation in mice maintained on anti-oxidant diet.

## Results

### The hypersensitivity of TSC2-deficient cells to oxidative stress is associated with increased mTORC1 activity and decreased eIF2αP

**Fig. 1:**
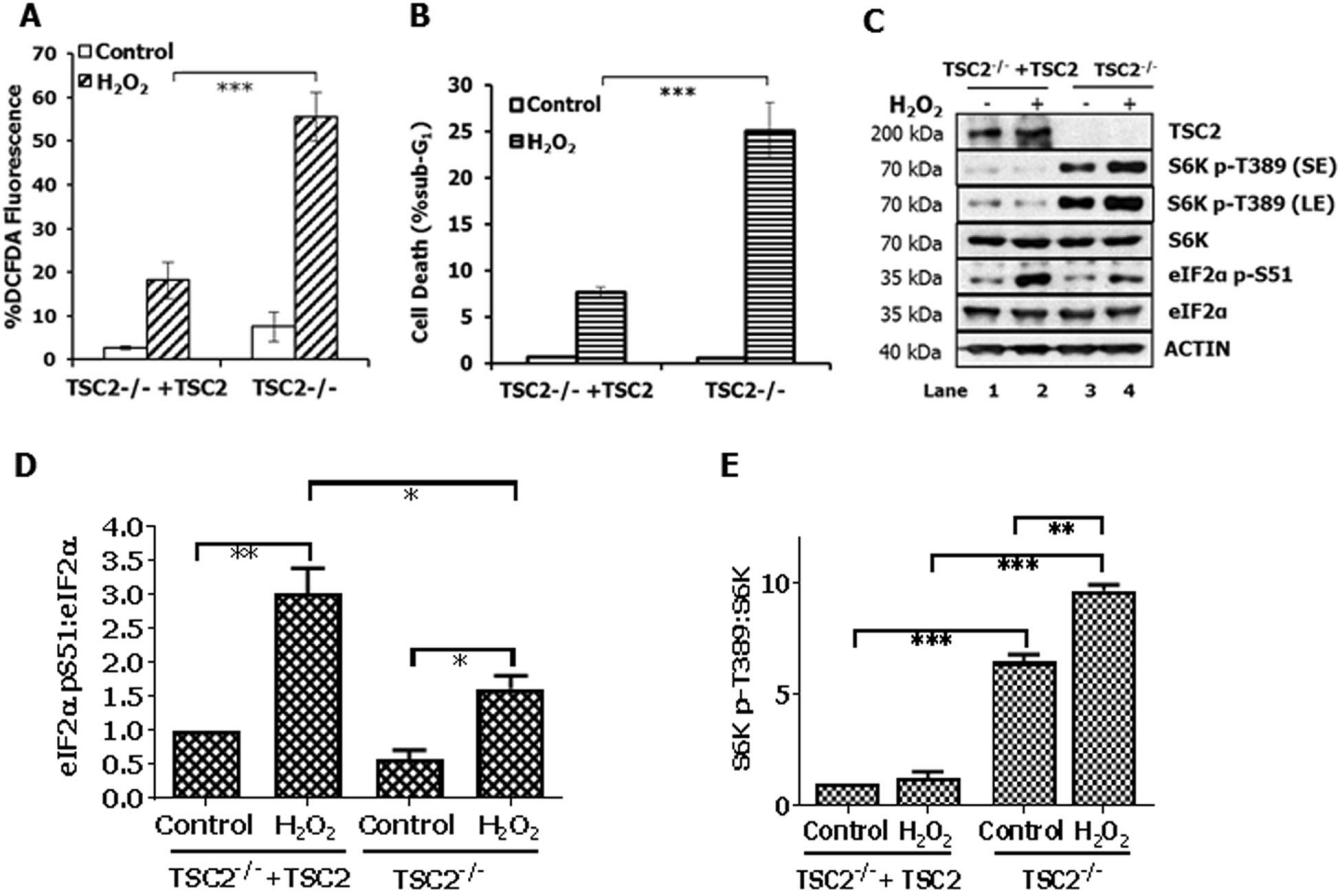
Increased mTORC1 activity and decreased eIF2αP in TSC2-deficient cells under oxidative stress. **a**TSC2-proficient (TSC2^−/−^+TSC2) and TSC2-deficient (TSC2^−/−^) MEFs were treated with 0.1 mM H_2_O_2_ for 45 min followed by the addition of 5 µM DCFDA with for 25 min and detection of ROS levels by FACS analysis. **b** TSC2-proficient and TSC2-deficient MEFs were treated with 2 mM H_2_O_2_ for 8 h. The percentage of cell death was determined by measuring the population of cells in sub-G_1_ by propidium iodide staining and FACS analysis. **a**, **b** Histograms represent the mean of 3 independent experiments and the error bars indicate the SEM. ***p < 0.005. **c** TSC2-proficient or TSC2-deficient MEFs were treated with 0.5 mM H_2_O_2_ for 2 h. Protein extracts (50 µg) were immunoblotted for the indicated proteins. **d**, **e** Quantification of that ratio of eIF2α-pS51/eIF2α and S6K pT389/S6K from three separate experiments with cells and conditions shown in **c**. *p < 0.05 **p < 0.01 ***p < 0.005

TSC2^−/−^ mouse embryonic fibroblasts (MEFs) as well as TSC2^−/−^ MEFs reconstituted with TSC2 (herein referred to as TSC2-deficient and proficient cells, respectively^26^) were tested for their susceptibility to oxidative stress. Treatment with hydrogen peroxide (H_2_O_2_), which is a natural metabolite and mediator of growth factor signaling^1^, increased intracellular ROS production and the susceptibility of TSC2-deficient cells to death compared to TSC2-proficient cells (Fig. 1a, b). Treatment with H_2_O_2_ increased eIF2αP at a higher level in TSC2-proficient than in TSC2-deficient cells (Fig. 1c, d). Also, TSC2-deficient cells exhibited increased mTORC1 activity compared to proficient cells as indicated by the higher ribosomal S6 kinase (S6K) T389 phosphorylation in the former than latter cells (Fig. 1c, e). Treatment with H_2_O_2_ further increased S6K T389 phosphorylation in TSC2-deficient cells compared to proficient cells indicating the hyper-activation of mTORC1 in these cells under oxidative stress (Fig. 1c, e). These data provided a link between mTORC1 and eIF2αP in ROS production and susceptibility of TSC2-deficient cells to oxidative stress.

### PERK activity is impaired in TSC2-deficient cells under oxidative stress

**Fig. 2:**
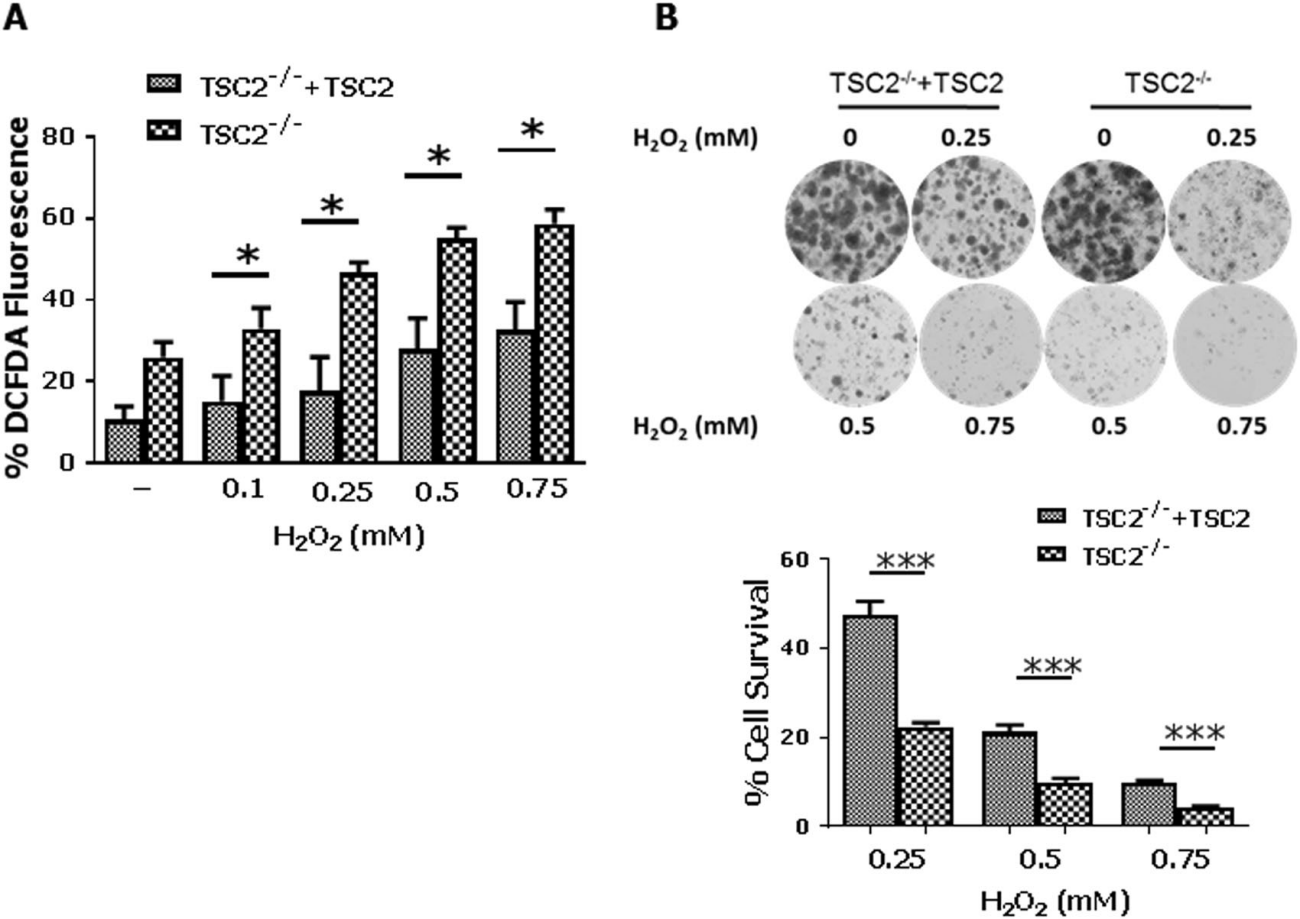
Increased ROS production is associated with increased susceptibility of TSC2-deficient cells to death. **a** TSC2-proficient (TSC2^−/−^ + TSC2) and TSC2-deficient (TSC2^−/−^) MEFs were treated with the indicated concentrations of H_2_O_2_ for 45 min followed by the addition of 5 µM DCFDA for an additional 25 min. Detection of ROS levels in cells was assessed by FACS analysis. Histograms represent the mean of 3 independent experiments and the error bars indicate SEM. *p < 0.05 ***p < 0.005. **b** TSC2-proficient and TSC2-deficient MEFs (2 × 10^3^) were treated with the indicated amounts of H_2_O_2_ for 8 h. Media was refreshed every second day for one week. Cells were fixed in formalin and stained with crystal violet (top panel). Cell survival was assessed by extraction in 10% acetic acid and detection of optical density (OD) at 570 nM (bottom panel). Histogram represent the mean of two independent experiments done in quadruplicate and the error bars indicate SEM. *p < 0.05 ***p < 0.005

**Fig. 3:**
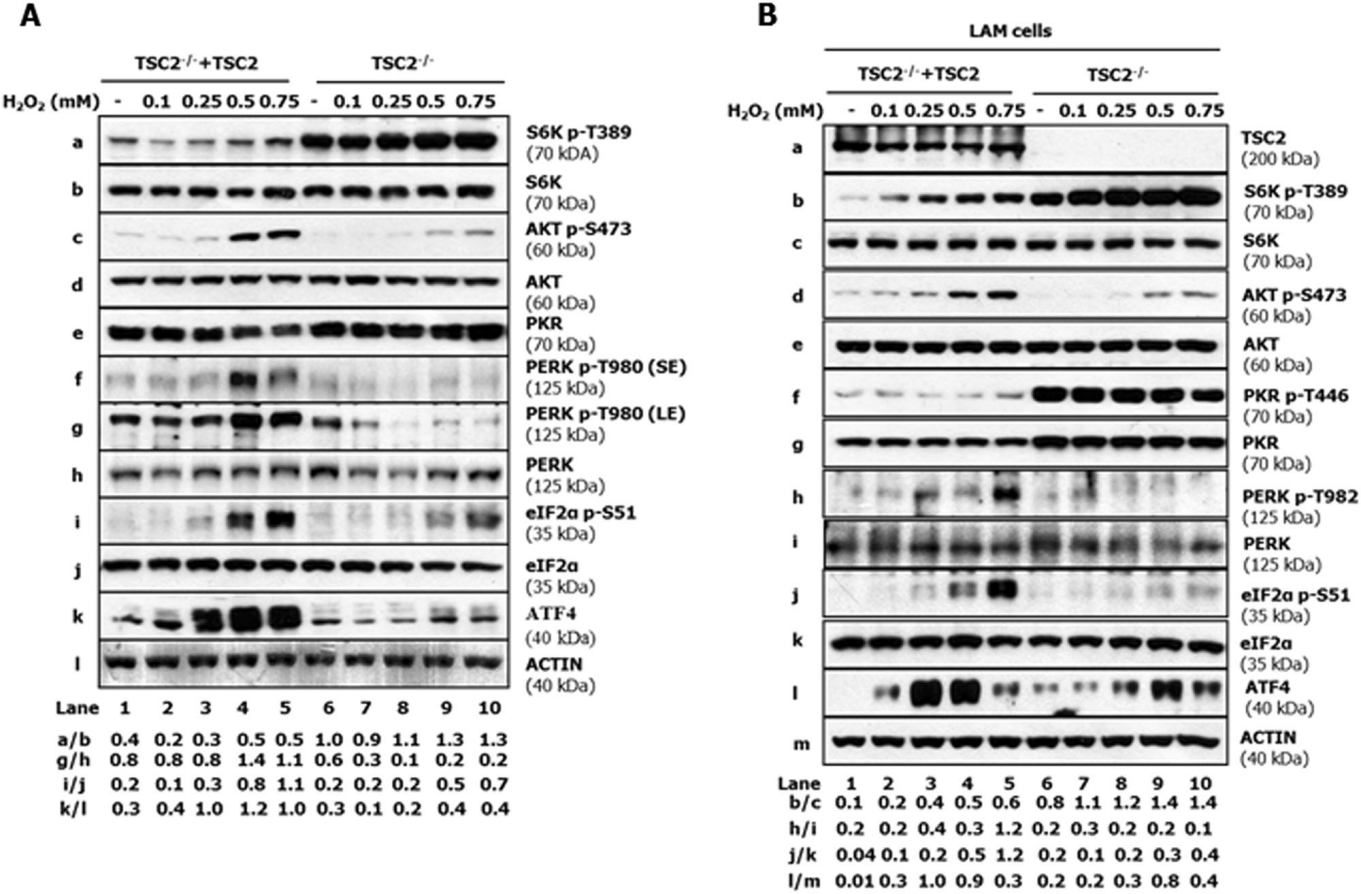
Impaired PERK activity, eIF2αP and ATF4 expression in TSC2-deficient cells under oxidative stress. TSC2-proficient and TSC2-deficient MEFs (**a**) or LAM cells (**b**) were treated with the indicated concentrations of H_2_O_2_ for 2 h. Protein extracts (50 µg) were immunoblotted for the indicated proteins. Blots are representatives of three (**a**) or two (**b**) biological replicates. The ratios of phosphorylated to total proteins for the representative blots are shown. LE long exposure of blots, SE short exposure of blots

Considering that an equal amount of H_2_O_2_ resulted in different ROS levels between TSC2-proficient and TSC2-deficient MEFs (Fig. 1a), we investigated whether the differences in mTORC1 activity and eIF2αP between the two cell types were an effect of variable ROS production. To this end, ROS levels were assessed in these cells subjected to increasing concentrations of H_2_O_2_. The results confirmed that ROS production was higher in TSC2-deficient than proficient MEFs in response to equal amounts of H_2_O_2_ (Fig. 2a). Colony formation assays showed that the survival of both cell types was inversely proportional to the amount of H_2_O_2_ treatment (Fig. 2b). Nevertheless, TSC2-proficient MEFs displayed a better survival than TSC2-deficient MEFs in response to treatments with an equal amount of H_2_O_2_ (Fig. 2b). Treatment with increasing concentrations of H_2_O_2_ resulted in a higher activation of PERK by autophosphorylation at T980 in TSC2-proficient than deficient MEFs (Fig. 3a). Also, eIF2αP was induced at a higher level in TSC2-proficient than in deficient MEFs in response to increasing concentrations of H_2_O_2_ (Fig. 3a). Moreover, phosphorylation of S6K T389 by mTORC1 was increased at a higher level in TSC2-deficient than in proficient MEFs whereas AKT S473 phosphorylation was substantially reduced in TSC2-deficient compared to proficient MEFs under the pro-oxidant treatments (Fig. 3a).

The above data indicated an increased susceptibility of TSC2-deficient MEFs compared to proficient MEFs, when both cell types were exposed to equal concentrations of H_2_O_2_. However, when cells were treated with concentrations of H_2_O_2_ that produced equivalent ROS levels (e.g., 0.5 mM for TSC2-proficient and 0.25 mM for TSC2-deficient MEFs), both cell types were similarly tolerant to oxidative stress (Fig. 2b). Nevertheless, equal ROS levels resulted in reduced PERK activity and decreased eIF2αP in TSC2-deficient compared to proficient MEFs (Fig. 3a, compare lanes 4 and 8). This data suggested that decreased PERK activity and eIF2αP was not an effect of variable ROS production between the two cell types.

We also examined the signaling effects of oxidative stress in human lymphangioleiomyomatosis (LAM) 621–101 cells that were deficient in TSC2^27^. Treatment with H_2_O_2_ was associated with an inhibition of PERK T982 phosphorylation and decreased eIF2αP in TSC2-deficient LAM cells compared to LAM cells reconstituted with human TSC2 (Fig. 3b). As with MEFs, AKT S473 phosphorylation was substantially lower in TSC2-deficient than proficient LAM cells in either the absence or presence of H_2_O_2_ (Fig. 3b). TSC2-deficient LAM cells displayed increased levels of PKR protein (Fig. 3b), an effect that was also evident but to a lesser extent in TSC2-deficient MEFs (Fig. 3a). Activation of the eIF2α kinase PKR, which has been linked to oxidative stress^28^, by autophosphorylation at T446 was increased in TSC2-deficient compared to proficient LAM cells as indicated by immunoblotting with an antibody specific for human PKR (Fig. 3b). However, exposure to increasing concentrations of H_2_O_2_ did not significantly alter PKR T446 phosphorylation in TSC2-deficient LAM cells as opposed to PERK T982 phosphorylation, which was gradually reduced (Fig. 3b). These data suggested that decreased eIF2αP is associated with the downregulation of PERK but not PKR in TSC2-deficient cells under oxidative stress.

### Reduced PERK activity and eIF2αP in TSC2-deficient cells under oxidative stress depends on mTORC1

**Fig. 4:**
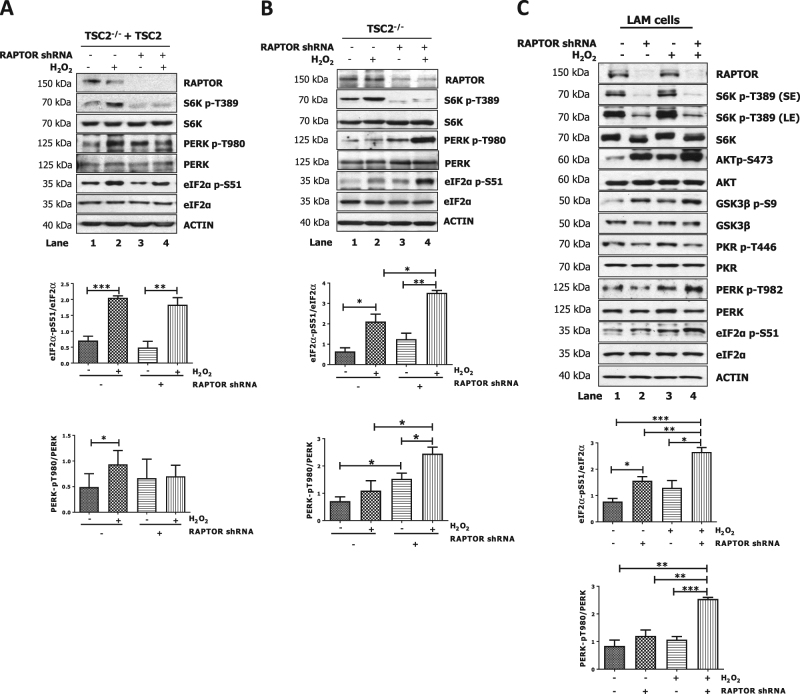
Hyper-activated mTORC1 mediates the reduction of PERK activity and eIF2αP in TSC2-deficient cells under oxidative stress. TSC2-proficient (**a**, TSC2^−/−^ + TSC2) or TSC2-deficient MEFs (**b**, TSC2^−/−^) were infected with pLKO lentiviruses lacking (−) or containing (+) an shRNA against mouse RAPTOR. **c** TSC2-deficient LAM cells were infected with pLKO lentiviruses lacking (−) or containing shRNA against human RAPTOR. **a**–**c** Cells were left untreated or treated with 0.5 mM H_2_O_2_ for 2 h. Protein extracts (50 μg) were immunoblotted for the indicated proteins. Quantifications of eIF2α-pS51/eIF2α and PERK-pT982/PERK from three biological replicates are shown. *p < 0.05 **p < 0.01 ***p < 0.005. LE long exposure of blots, SE short exposure of blots

Next, we determined whether mTORC1 plays a role in the inhibition of PERK and eIF2αP in TSC2-deficient cells under oxidative stress. To this end, cells were impaired for mTORC1 by infection with lentiviruses expressing RAPTOR shRNA. Downregulation of RAPTOR led to decreased S6K T389 phosphorylation in TSC2-proficient and TSC2-deficient MEFs in either the absence or presence of H_2_O_2_ (Fig. 4a,b). Loss of mTORC1 activity did not significantly affect PERK T980 phosphorylation or eIF2αP in TSC2-proficient MEFs prior or after H_2_O_2_ treatment (Fig. 4a). Downregulation of RAPTOR resulted in increased PERK T980 phosphorylation and eIF2αP in TSC2-deficient MEFs in the absence of H_2_O_2_ treatment, and it further increased PERK activity and eIF2αP in the same cells treated with H_2_O_2_ (Fig. 4b, compare lane 1 with 3 and lane 2 with 4). As with TSC2^−/−^ MEFs, PERK T982 phosphorylation and eIF2αP were increased at a higher level in H_2_O_2_-treated TSC2-deficient LAM cells impaired for RAPTOR compared to the same cells containing RAPTOR (Fig. 4c, compare lane 3 with 4). Downregulation of RAPTOR resulted in decreased PKR T446 phosphorylation in TSC2-deficient LAM cells in either the absence or presence H_2_O_2_ treatment (Fig. 4c, compare lane 1 with 2 and lane 3 with 4). This data indicated that mTORC1 increases PKR activity, which, however, is not subjected to further regulation by mTORC1 under oxidative stress. Also, RAPTOR downregulation led to a substantial increase in AKT S473 and glycogen synthase kinase 3β (GSK3β) S9 phosphorylation in TSC2-deficient LAM cells, which were further enhanced by H_2_O_2_ treatment (Fig. 4c, compare lane 1 with 3 and lane 2 with 4). The increased phosphorylation of AKT and GSK3β in TSC2-deficient cells impaired for RAPTOR can be explained by the alleviation of the negative effects of mTORC1 on PI3K-AKT signaling in these cells as previously shown^29–31^.

### eIF2αP in TSC2-deficient cells elicits pro-oxidant and pro-death effects in response to oxidative stress

**Fig. 5:**
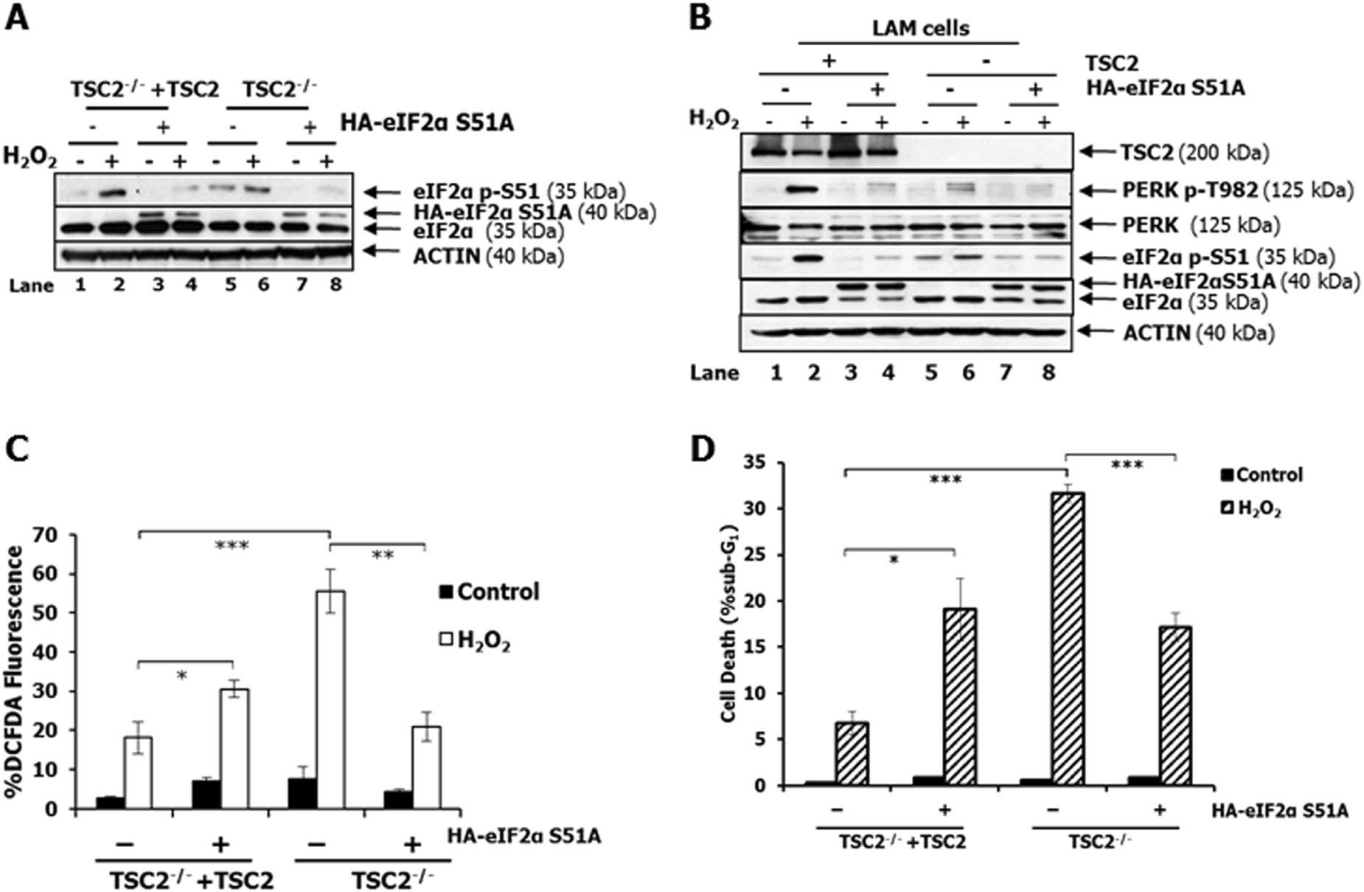
eIF2αP displays pro-oxidant and pro-death effects in TSC2-deficient cells. TSC2-proficient (TSC2^−/−^ + TSC2) and TSC2-deficient (TSC2^−/−^) MEFs (**a**) as well as TSC2-proficient and TSC2-deficient LAM cells (**b**) lacking or expressing HA-eIF2αS51A were left untreated or treated with 0.5 mM H_2_O_2_ for 2 h. Protein extracts (50 µg) were subjected to immunoblotting for the indicated proteins. **c** TSC2-proficient or TSC2-deficient MEFs either lacking or expressing HA-eIF2αS51A were treated with 0.1 mM of H_2_O_2_ for 45 min followed by the addition of 5 µM DCFDA for 25 min and detection of ROS by FACS analysis. **d** TSC2-proficient as well as TSC2-deficient MEFs either lacking or expressing HA-eIF2αS51A were treated with 2 mM H_2_O_2_ for 8 h. Cell death was determined by the percentage (%) of cells in sub-G_1_ after propidium iodide (PI) staining and FACS analysis. **c**, **d** Histograms represent the mean of three independent experiments and the error bars indicate SEM. *p < 0.05; **p < 0.01; ***p < 0.005

To determine the biological effects of eIF2αP in TSC2-deficient as well as proficient cells, we established cells expressing a hemagglutinin (HA)-tagged form of human eIF2α bearing the S51A mutation (HA-eIF2αS51A)^32^. Expression of HA-eIF2αS51A inhibited the phosphorylation of endogenous eIF2α in MEFs as well as in LAM cells in the absence or presence of H_2_O_2_ (Fig. 5a, b). Expression of HA-eIF2αS51A resulted in increased ROS production in TSC2-proficient MEFs and decreased ROS production in TSC2-deficient MEFs in response to H_2_O_2_ treatment (Fig. 5c). Also, HA-eIF2αS51A expression rendered TSC2-proficient MEFs increasingly susceptible to H_2_O_2_-mediated death as opposed to TSC2-deficient MEFs, which became increasingly resistance to oxidative stress in the presence of HA-eIF2αS51A (Fig. 5d). These data indicated that further reduction of eIF2αP in TSC2-deficient cells elicits anti-oxidant and pro-survival effects in response to extrinsic oxidative stress. The data supported a pro-oxidant and pro-death role of eIF2αP in TSC2-deficient cells as opposed to TSC2-proficient cells, in which eIF2αP exhibits anti-oxidant and pro-survival effects.

### eIF2αP in TSC2-deficient cells mediates anti-tumor effects, which are alleviated by an anti-oxidant diet

**Fig. 6:**
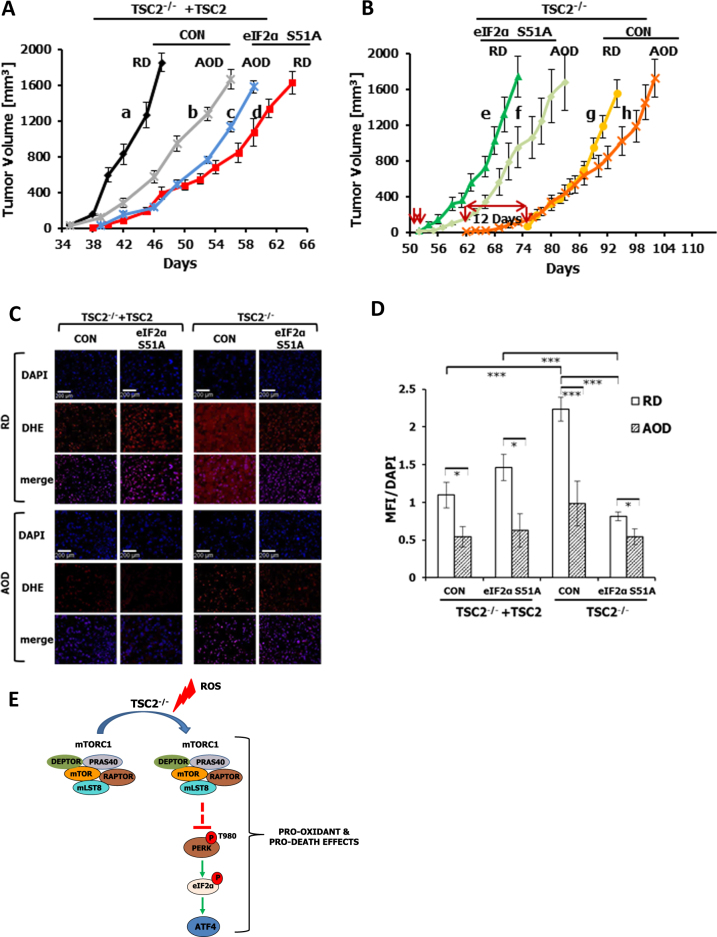
eIF2αP suppresses TSC2-deficient tumor initiation in mice under pro-oxidant conditions. **a**, **b** TSC2-proficient (**a**) or TSC2-deficient MEFs cells (**b**) either lacking (CON) or expressing HA-eIF2αS51A were transplanted in SCID Beige mice, which were kept on regular diet (RD) or an anti-oxidant diet (AOD). Each group consisted of five female 8-week-old SCID Beige mice, and each mouse received two subcutaneous injections of 2 × 10^6^ cells per site. Tumor initiation was monitored by palpation whereas tumor growth was assessed by measurements of the average volume of *n* = 5 × 2 = 10 tumors per group. **c** Tumor sections from mice under regular diet (RD) or the anti-oxidant diet (AOD) injected with TSC2-proficient or deficient MEFs lacking (control; CON) or expressing HA-eIF2αS51A. Tumor sections were used to detect ROS levels by dihydroethidium (DHE) staining whereas cell nuclei were visualized by 4’,6-diamidino-2-phenylindole (DAPI) staining. **d** DHE and DAPI staining in C was quantified by the ImageJ software. Histograms represent the average mean fluorescence intensity (MFI) of ROS levels normalized to DAPI measured in 10 images obtained from tumors grown in three separate mice per group. The error bars indicate SEM. *p < 0.05; **p < 0.01; ***p < 0.005. **e** Model of eIF2αP function in TSC2-deficient cells under oxidative stress. TSC2 deficiency leads to hyper-activation of mTORC1, which under conditions of increased ROS levels results in the inactivation of PERK. PERK inactivation by mTORC1 is most likely indirect and may involve the action of protein phosphatases implicated in mTORC1 signaling (see text). Downregulation of PERK activity and eIF2αP leads to decreased expression of ATF4, which can disarm anti-oxidant responses and contribute to increased susceptibility of TSC2-deficient cells to oxidative insults. The low levels of eIF2αP in TSC2-deficient cells can establish conditions that favor the inhibition of tumor formation in mice, an effect that is significantly alleviated in mice kept on an anti-oxidant diet

We further assessed the role of eIF2αP in tumor formation of TSC2-deficient cells in mice based on previous work showing that the TSC2-deficient MEFs employed in our study are tumorigenic in immunocompromised mice^33^. Transplantation assays in severe combined immunodeficiency (SCID) Beige mice, which were maintained under a regular diet (RD), showed that TSC2-deficient cells developed tumors ~40 days later than TSC2-proficient cells (Fig. 6a, b; compare graph a with g). The increased tumorigenic potency of TSC2-proficient compared to TSC2-deficient MEFs was previously reported and attributed to better responses of the former cells to growth factors due to functional PI3K-AKT signaling^33^. In addition to accelerated tumor initiation, TSC2-proficient tumors exhibited better growth rates than the TSC2-deficient tumors (Fig. 6a, b; compare graphs a and g). TSC2-proficient tumors with impaired eIF2αP (i.e. HA-eIF2αS51A-expressing cells) exhibited a minor delay in initiation (~4 days) but a significant reduction in growth rates compared to control TSC2-proficient tumors with intact eIF2αP (Fig. 6a; compare graphs a and d). On the other hand, TSC2-deficient tumors with impaired eIF2αP displayed accelerated initiation by ~23 days compared to TSC2-deficient control tumors whereas the rates of tumor growth did not significantly differ between the two cell types (Fig. 6b; compare graphs e and h) (e and g). These data suggested that eIF2αP facilitates the growth of TSC2-proficient tumors but inhibits the initiation of TSC2-deficient tumors in SCID mice.

We next examined whether inhibition of tumor initiation of TSC2-deficient cells by eIF2αP is affected by pro-oxidant conditions in the tumor microenvironment. To address this matter, TSC2-proficient and TSC2-deficient MEFs transplanted in SCID mice were kept on an anti-oxidant diet (AOD) consisting of N-acetylcysteine in the drinking water and DL-α-tocopheryl acetate (vitamin E) in the food^34^. Treatment of mice with AOD did not affect the initiation of TSC2-proficient tumors but had a substantial inhibitory effect on tumor growth (Fig. 6a; compare graphs a and b). Tumor growth inhibition in mice subjected to AOD could be explained by previous findings supporting a stimulatory effect of oxidative stress on tumor angiogenesis and growth^35^. Also, AOD did not affect the initiation of TSC2-proficient tumors with impaired eIF2αP but exhibited a modest stimulatory effect on their growth rate (Fig. 6a; compare graphs c and d). When mice were transplanted with TSC2-deficient cells, AOD accelerated the initiation of tumors with intact eIF2αP (control) by 12 days but had no effect on the initiation of tumors with impaired eIF2αP (Fig. 6b, compare graphs g and h as well as graphs e and f, respectively). Also, the difference in tumor initiation between TSC2-deficient MEFs with intact (control) or impaired eIF2αP decreased from 23 days under RD to 10 days under AOD (Fig. 6b, compare graphs e and g with graphs f and h, respectively). Collectively, the data suggested that a significant fraction (~50%) of the anti-tumor effects of eIF2αP on TSC2-deficient cells in mice is alleviated by the anti-oxidant diet.

To confirm the anti-oxidant effects of AOD in mice, ROS production was assessed in tumor sections with the dihydroethidium (DHE) fluorescence method^36^. TSC2-proficient tumors with impaired eIF2αP from RD-treated mice exhibited increased ROS levels compared to TSC2-proficient tumors with intact eIF2αP consistent with an anti-oxidant function of eIF2αP in these tumors (Fig. 6c, d). On the other hand, TSC2-deficient tumors with impaired eIF2αP from RD-treated mice displayed decreased ROS levels compared to TSC2-deficient tumors with intact eIF2αP supporting a pro-oxidant function of eIF2αP in these tumors (Fig. 6c, d). TSC2-proficient tumors with intact eIF2αP from RD-treated mice exhibited decreased ROS levels compared to TSC2-deficient tumors indicating increased ROS production in tumors lacking TSC2 (Fig. 6c, d). When mice subjected to AOD, ROS levels were substantially reduced in TSC2-proficient as well as deficient tumors (Fig. 6c, d). Also, AOD treatment had a stronger effect on the inhibition of ROS levels in TSC2-deficient than TSC2-proficient tumors with intact eIF2αP. Moreover, inhibition of ROS synthesis by AOD was more evident in TSC2-proficient than deficient tumors with impaired eIF2αP (i.e., HA-eIF2αS51A-expressing tumors). These data suggested that eIF2αP mediates anti-oxidant effects in TSC2-proficient tumors and pro-oxidant effects in TSC2-deficient tumors.

## Discussion

We found that TSC2-deficient cells are impaired in the activation of PERK and eIF2αP under oxidative stress, a process that is associated with an increased susceptibility of these cells to oxidative insults (see model in Fig. 6e). We also found that increased mTORC1 activity is responsible for the inhibition of PERK activity in TSC2-deficient cells under oxidative stress. Hyper-activated mTORC1 is unlikely to exert a direct effect on PERK in TSC2-deficient cells because mass spectrometry data did not identify PERK as a substrate of mTOR^37, 38^. A plausible explanation is that PERK inactivation is mediated by a phosphatase, whose activity is induced by oxidative stress in TSC2-deficient cells. Previous data implicated protein phosphatase (PP) 2A activity in the regulation of mTOR signaling in cells with increased oxidative stress^7^. Contrary to PERK, PKR protein levels and activity are increased in TSC2-deficient cells, which, however, could not account for the inhibition of eIF2αP in response to oxidative insults. TSC2-deficient cells exhibit increased signal transducer and activator of transcription (STAT) signaling^39^, which can increase PKR levels at the transcriptional level^40^.

Apart from PERK, GCN2, and HRI may play a role in the regulation of eIF2αP in TSC2-deficient cells. Specifically, pharmacological inhibition of mTORC1 leads to GCN2 activation and increased eIF2αP in a mechanism dependent on the catalytic subunit of PP6C^41^. Interestingly, eIF2αP is impaired in TSC2-deficient MEFs under stress that causes GCN2 activation such as leucine deprivation or treatment with the histidinyl-tRNA synthetase inhibitor histidinol^41^. In *Saccharomyces cerevisiae*, TOR inhibits GCN2 activity through TAP42, a regulator of type 2A-related phosphatase^42^, whereas in *Schizosaccharomyces pombe*, Tor2, as part of mTORC1, prevents GCN2 activation and eIF2αP in the presence of nitrogen and amino acids^43^. Other studies showed that exposure of *S. pombe* to oxidative stress results in the activation of GCN2 and HRI 2, which is one of the two eIF2α kinases in fission yeast related to mammalian HRI^44, 45^. In mammalian cells, HRI is activated by oxidative stress and plays an important role in the reduction of oxidative stress in mice during iron deficiency^46^.

mTORC1 may decrease eIF2αP in TSC2-deficient cells through additional mechanisms based on its ability to phosphorylate the β subunit of eIF2, which promotes eIF2α dephosphorylation by PP1^47^. Decreased eIF2αP may act in a feedback loop to increase mTORC1 activity in TSC2-deficient cells based on previous findings showing an inhibitory effect of increased eIF2αP on mTORC1 activity in cells subjected to pro-oxidant treatments^24^. Inhibition of mTORC1 by eIF2αP can take place in cells under different forms of stress via increased expression of Sestrin-2 or protein regulated in development and DNA damage response (REDD1), both of which inhibit mTORC1 and are transcriptionally upregulated by ATF4^48–52^.

Considering the key role of the PERK-eIF2αP-ATF4 axis in anti-oxidant responses^19, 20^, its downregulation may account for the increased susceptibility of TSC2-deficient cells to pro-oxidant treatments. Also, decreased eIF2αP delays the initiation of tumor formation of TSC2-deficient cells in immune deficient mice, an effect that is significantly alleviated in mice kept on an anti-oxidant diet. These finds provide a potentially important link between the pro-oxidant function of eIF2αP and inhibition of TSC2-deficient tumor formation in mice. Increased ROS production can promote tumor formation but can also render tumors susceptible to further increase of oxidative stress^53^. Oxidative stress in tumors can derive from intrinsic as well as extrinsic sources like tumor-associated fibroblasts or macrophages, which synergize in the induction of a pro-oxidant environment^54^. What determines death over survival by eIF2αP in TSC2-deficient cells is not clear but the presence of functional TSC may establish a threshold of eIF2αP above which cells display increased tolerance and survival to oxidative stress. In the absence of TSC function, eIF2αP levels below the threshold may render cells increasingly vulnerable to oxidative stress.

Our study addresses the cell-autonomous effects of eIF2αP on TSC2-deficient tumor formation in immune deficient mice. However, eIF2αP may also control TSC2-deficient tumor formation via immune-regulatory pathways. Specifically, eIF2αP in tumors has been linked to the induction of immunogenic cell death (ICD)^55^, which requires the activation of immune-mediated anti-tumor responses in the tumor bed in response to certain chemotherapeutic drugs^56^. A recent study showed that increased ROS in TSC-proficient tumor cells under hydrostatic pressure activates the PERK-eIF2αP arm and promotes ICD^57^. Thus, the effects of PERK and eIF2αP on tumor formation under pro-oxidant conditions may engage both cell-autonomous and immune-regulatory mechanisms. Considering the ability of an anti-oxidant diet to accelerate the formation of TSC2-deficient tumors in mice, implementation of pro-oxidant diets may have the potential to prevent or delay the development of TSC2-deficient cancer. For example, ascorbic acid (vitamin C) can suppress ovarian and colorectal tumors in immune deficient mice through an increase in oxidative stress, when it is administered parenterally and/or at pharmacological doses^58–60^. Our work raises the interesting hypothesis that vitamin C and possibly other pro-oxidant dietary supplements could be beneficial for the treatment of TSC-deficient tumors.

## Materials and methods

### Cell culture and treatments

The generation and culture of TSC2-proficient and TSC2-deficient MEFs was previously described^26^. The origin of the LAM 621–101 cells was previously described^27^. The generation of TSC2-proficient LAM 621–101 cells was performed by infection with pBABE retroviruses containing a FLAG-tagged form of human TSC2 and selection of cells in 200 µg/mL hygromycin (BioShop Canada). The origin and preparation of viruses expressing shRNAs against mouse or human RAPTOR was previously described^32, 61^. Generation of eIF2αP-deficient TSC2 MEFs and LAM 621–101 cells was described^32^. Colony formation assays of cells subjected to oxidative stress were performed as previously described^20^.

### Protein extraction and immunoblotting

Protein extraction and immunoblot analyses were performed as described previously^32^. The phosphorylation of PERK, AKT, S6K, ATF4, GSK3 or eIF2α was tested in parallel by loading equal amounts of protein extracts (50 µg) from the same set of experiments on two identical SDS-10% polyacrylamide gels. After transfer of proteins, the two identical blots were cut into different pieces based on the size of proteins to be tested. One piece of the blot was probed for the phosphorylated protein of interest whereas the corresponding piece of the replica blot for total protein. The antibodies were obtained from Cell Signaling Technology unless otherwise indicated. The antibodies used were as follows: rabbit monoclonal against phosphorylated eIF2α at S51 (Novus Biologicals), mouse monoclonal against eIF2α, mouse monoclonal against PERK, rabbit monoclonal against phosphorylated S6K at T389, rabbit monoclonal against S6K, rabbit monoclonal against RAPTOR, rabbit monoclonal against phosphorylated AKT S473 or AKT, mouse monoclonal against mouse PKR (B-10; Santa Cruz Biotech.), rabbit monoclonal against human PKR phosphorylated at T446 (clone E120; Abcam), mouse monoclonal against human PKR (clone 13B8-F9;^62^), mouse monoclonal antibody to actin (ICN Biomedicals Inc.), rabbit polyclonal against ATF4 (Proteintech), rabbit monoclonal against TSC2, rabbit polyclonal against GSK3β S9. The rabbit monoclonal against phosphorylated PERK at T982 was previously described^32^. All antibodies were used at a final concentration of 0.1–1 μg/ml. Anti-mouse or anti-rabbit IgG antibodies conjugated to horseradish peroxidase (HRP) (Mandel Scientific) were used as secondary antibodies (1 × 1000 dilution) and proteins were visualized with enhanced chemiluminescence (ECL) (Thermo Scientific). Protein bands were quantified by the ImageJ software in the linear range of exposure.

### Xenograft tumor assays

The xenograft tumor assays were performed as described^20^. Specifically, 8-week-old SCID Beige female mice (Charles River Inc.) were subjected to bilateral subcutaneous thigh injections with either TSC2-proficient or deficient MEFs. For each set of the experiment 5 mice were used and each mouse received 2 × 10^6^ cells per injected site. Mice were fed on standard chow (#2918, Envigo) lacking (RD) or supplemented with 500 IU/g of DL-alpha-tocopheryl acetate (vitamin E) (Envigo) and 1 g/L N-acetylcysteine in the drinking water (AOD)^34^. Following tumor transplantation, mice were monitored for signs of pain or discomfort three times per week. Tumor initiation was detected by palpation whereas tumor growth was measured by digital calipers, and tumor volume was calculated by the formula: tumor volume (mm^3^) = 1/2 × (length (mm)) × (width (mm))^2^. The animal studies were performed in accordance with the Institutional Animal Care and Use Committee (IACUC) of McGill University and procedures were approved by the Animal Welfare Committee of McGill University (protocol #5754).

### Flow cytometry analysis

Cell death analysis by flow cytometry was previously described^32^. ROS levels were quantified using 2’,7’-dichlorodihydrofluorescein diacetate (DCFDA, Molecular Probes) as reported^24^. Data were analyzed using the FlowJo software (Tree Star, Inc.).

### Detection of ROS in tissue sections

ROS production was evaluated by the fluorescent probe dihydroethidium (DHE; Sigma)^36^. Formalin-fixed, paraffin-embedded 5 µm thick tissue sections were de-paraffinised, treated with xylene, rehydrated through a graded alcohol series and rinsed in deionized water. Sections were then incubated with 4 µM of DHE for 30 min in a dark humidified chamber at 37 °C. Visualization of red fluorescence produced by the oxidation of DHE by free radicals was performed on a Zeiss M1 fluorescence microscope and signals were quantified by the ImageJ software.

### Statistical analysis

Error bars represent standard error as indicated and significance in differences between arrays of data tested was determined using two-tailed Student T test (Microsoft Excel).

## References

[1] Holmstrom KM, Finkel T (2014). Cellular mechanisms and physiological consequences of redox-dependent signalling. Nat. Rev. Mol. Cell. Biol..

[2] Landriscina M, Maddalena F, Laudiero G, Esposito F (2009). Adaptation to oxidative stress, chemoresistance, and cell survival. Antioxid. Redox. Signal..

[3] Nemoto S, Finkel T (2002). Redox regulation of forkhead proteins through a p66shc-dependent signaling pathway. Science.

[4] Nogueira V, Hay N (2013). Molecular pathways: reactive oxygen species homeostasis in cancer cells and implications for cancer therapy. Clin. Cancer Res..

[5] Manning BD, Toker A (2017). AKT/PKB Signaling: Navigating the Network. Cell.

[6] Laplante M, Sabatini DM (2012). mTOR signaling in growth control and disease. Cell.

[7] Li M (2010). Multi-mechanisms are involved in reactive oxygen species regulation of mTORC1 signaling. Cell Signal..

[8] Tee AR, Manning BD, Roux PP, Cantley LC, Blenis J (2003). Tuberous sclerosis complex gene products, tuberin and hamartin, control mTOR signaling by acting as a GTPase-activating protein complex toward Rheb. Curr. Biol..

[9] Inoki K, Li Y, Xu T, Guan KL (2003). Rheb GTPase is a direct target of TSC2 GAP activity and regulates mTOR signaling. Genes Dev..

[10] Dibble CC (2012). TBC1D7 is a third subunit of the TSC1-TSC2 complex upstream of mTORC1. Mol. Cell..

[11] Thedieck K (2013). Inhibition of mTORC1 by astrin and stress granules prevents apoptosis in cancer cells. Cell.

[12] Zhang J (2013). A tuberous sclerosis complex signalling node at the peroxisome regulates mTORC1 and autophagy in response to ROS. Nat. Cell Biol..

[13] Grant CM (2011). Regulation of translation by hydrogen peroxide. Antioxid. Redox. Signal..

[14] Koromilas AE (2015). Roles of the translation initiation factor eIF2alpha serine 51 phosphorylation in cancer formation and treatment. Biochim. Biophys. Acta.

[15] Wek RC, Jiang HY, Anthony TG (2006). Coping with stress: eIF2 kinases and translational control. Biochem. Soc. Trans..

[16] Davies KJA (2016). Adaptive homeostasis. Mol. Asp. Med..

[17] Pakos‐Zebrucka K (2016). The integrated stress response. EMBO Rep..

[18] Back SH (2009). Translation attenuation through eIF2alpha phosphorylation prevents oxidative stress and maintains the differentiated state in beta cells. Cell Metab..

[19] Harding HP (2003). An integrated stress response regulates amino acid metabolism and resistance to oxidative stress. Mol. Cell.

[20] Rajesh K (2013). eIF2alpha phosphorylation bypasses premature senescence caused by oxidative stress and pro-oxidant antitumor therapies. Aging.

[21] Ameri K, Harris AL (2008). Activating transcription factor 4. Int. J. Biochem. Cell. Biol..

[22] Shimizu Y, Hendershot LM (2009). Oxidative folding: cellular strategies for dealing with the resultant equimolar production of reactive oxygen species. Antioxid. Redox. Signal..

[23] Venditti P, Di SL, Di MS (2013). Mitochondrial metabolism of reactive oxygen species. Mitochondrion.

[24] Rajesh K (2015). Phosphorylation of the translation initiation factor eIF2alpha at serine 51 determines the cell fate decisions of Akt in response to oxidative stress. Cell Death Dis..

[25] Mounir Z (2011). Akt determines cell fate through inhibition of the PERK-eIF2{alpha} phosphorylation pathway. Sci. Signal..

[26] Huang J, Dibble CC, Matsuzaki M, Manning BD (2008). The TSC1-TSC2 complex is required for proper activation of mTOR complex 2. Mol. Cell. Biol..

[27] Yu J, Henske EP (2010). mTOR activation, lymphangiogenesis, and estrogen-mediated cell survival: the “perfect storm” of pro-metastatic factors in LAM pathogenesis. Lymphat. Res. Biol..

[28] Li G, Scull C, Ozcan L, Tabas I (2010). NADPH oxidase links endoplasmic reticulum stress, oxidative stress, and PKR activation to induce apoptosis. J. Cell Biol..

[29] Harrington LS (2004). The TSC1-2 tumor suppressor controls insulin-PI3K signaling via regulation of IRS proteins. J. Cell Biol..

[30] Shah OJ, Wang Z, Hunter T (2004). Inappropriate activation of the TSC/Rheb/mTOR/S6K cassette induces IRS1/2 depletion, insulin resistance, and cell survival deficiencies. Curr. Biol..

[31] Manning BD (2005). Feedback inhibition of Akt signaling limits the growth of tumors lacking Tsc2. Genes Dev..

[32] Tenkerian C (2015). mTORC2 Balances AKT Activation and eIF2alpha Serine 51 Phosphorylation to Promote Survival under Stress. Mol. Cancer Res..

[33] Zhang H (2007). PDGFRs are critical for PI3K/Akt activation and negatively regulated by mTOR. J. Clin. Invest..

[34] Sayin VI (2014). Antioxidants accelerate lung cancer progression in mice. Sci. Transl. Med..

[35] Kim YW, Byzova TV (2014). Oxidative stress in angiogenesis and vascular disease. Blood.

[36] Saenz-de-Viteri M (2014). Oxidative stress and histological changes in a model of retinal phototoxicity in rabbits. Oxid. Med. Cell. Longev..

[37] Hsu PP (2011). The mTOR-regulated phosphoproteome reveals a mechanism of mTORC1-mediated inhibition of growth factor signaling. Science.

[38] Yu Y (2011). Phosphoproteomic analysis identifies Grb10 as an mTORC1 substrate that negatively regulates insulin signaling. Science.

[39] El Hashemite N, Zhang H, Walker V, Hoffmeister KM, Kwiatkowski DJ (2004). Perturbed IFN-gamma-Jak-signal transducers and activators of transcription signaling in tuberous sclerosis mouse models: synergistic effects of rapamycin-IFN-gamma treatment. Cancer Res..

[40] Ward SV, Markle D, Das S, Samuel CE (2002). The promoter-proximal KCS element of the PKR kinase gene enhances transcription irrespective of orientation and position relative to the ISRE element and is functionally distinct from the KCS-like element of the ADAR deaminase Promoter. J. Interferon Cytokine Res..

[41] Wengrod J (2015). Phosphorylation of eIF2alpha triggered by mTORC1 inhibition and PP6C activation is required for autophagy and is aberrant in PP6C-mutated melanoma. Sci. Signal..

[42] Cherkasova VA, Hinnebusch AG (2003). Translational control by TOR and TAP42 through dephosphorylation of eIF2alpha kinase GCN2. Genes Dev..

[43] Valbuena N, Rozalen AE, Moreno S (2012). Fission yeast TORC1 prevents eIF2alpha phosphorylation in response to nitrogen and amino acids via Gcn2 kinase. J. Cell Sci..

[44] Zhan K (2002). Phosphorylation of eukaryotic initiation factor 2 by heme-regulated inhibitor kinase-related protein kinases in Schizosaccharomyces pombe is important for fesistance to environmental stresses. Mol. Cell. Biol..

[45] Zhan K, Narasimhan J, Wek RC (2004). Differential activation of eIF2 kinases in response to cellular stresses in Schizosaccharomyces pombe. Genetics.

[46] Zhang S (2018). HRI coordinates translation by eIF2alphaP and mTORC1 to mitigate ineffective erythropoiesis in mice during iron deficiency. Blood.

[47] Gandin V (2016). mTORC1 and CK2 coordinate ternary and eIF4F complex assembly. Nat. Commun..

[48] Ye J (2015). GCN2 sustains mTORC1 suppression upon amino acid deprivation by inducing Sestrin2. Genes Dev..

[49] Nikonorova IA (2017). Obesity challenges the hepatoprotective function of the integrated stress response to asparaginase exposure in mice. J. Biol. Chem..

[50] Kim HJ (2017). Carbon monoxide protects against hepatic steatosis in mice by inducing sestrin-2 via the PERK-eIF2alpha-ATF4 pathway. Free Radic. Biol. Med.

[51] Parmigiani A (2014). Sestrins inhibit mTORC1 kinase activation through the GATOR complex. Cell Rep..

[52] Dennis MD, Coleman CS, Berg A, Jefferson LS, Kimball SR (2014). REDD1 enhances protein phosphatase 2A-mediated dephosphorylation of Akt to repress mTORC1 signaling. Sci. Signal..

[53] Schumacker PT (2015). Reactive oxygen species in cancer: a dance with the devil. Cancer Cell.

[54] Catalano V (2013). Tumor and its microenvironment: a synergistic interplay. Semin. Cancer Biol..

[55] Kepp O (2015). eIF2alpha phosphorylation as a biomarker of immunogenic cell death. Semin. Cancer Biol..

[56] Kroemer G, Galluzzi L, Kepp O, Zitvogel L (2013). Immunogenic cell death in cancer therapy. Annu. Rev. Immunol..

[57] Moserova I (2017). Caspase-2 and oxidative stress underlie the immunogenic potential of high hydrostatic pressure-induced cancer cell death. OncoImmunology.

[58] Chen Q (2008). Pharmacologic doses of ascorbate act as a prooxidant and decrease growth of aggressive tumor xenografts in mice. Proc. Natl Acad. Sci. USA.

[59] Ma Y (2014). High-dose parenteral ascorbate enhanced chemosensitivity of ovarian cancer and reduced toxicity of chemotherapy. Sci. Transl. Med..

[60] Yun J (2015). Vitamin C selectively kills KRAS and BRAF mutant colorectal cancer cells by targeting GAPDH. Science.

[61] Peterson TR (2009). DEPTOR is an mTOR inhibitor frequently overexpressed in multiple myeloma cells and required for their survival. Cell.

[62] Cuddihy AR, Wong AH, Tam NW, Li S, Koromilas AE (1999). The double-stranded RNA activated protein kinase PKR physically associates with the tumor suppressor p53 protein and phosphorylates human p53 on serine 392 in vitro. Oncogene.

